# EPS mid-career prize 2018: Inference within episodic memory reflects pattern completion

**DOI:** 10.1177/1747021820959797

**Published:** 2020-10-08

**Authors:** Siti Nurnadhirah Binte Mohd Ikhsan, James A Bisby, Daniel Bush, David S Steins, Neil Burgess

**Affiliations:** 1 UCL Institute of Cognitive Neuroscience, University College London, London, UK; 2 Division of Psychiatry, University College London, London, UK; 3 UCL Institute of Neurology, University College London, London, UK

**Keywords:** Episodic memory, inference, pattern completion, recollection, hippocampus

## Abstract

Recollection of episodic memories is a process of reconstruction where coherent events are inferred from subsets of remembered associations. Here, we investigated the formation of multielement events from sequential presentation of overlapping pairs of elements (people, places, and objects/animals), interleaved with pairs from other events. Retrievals of paired associations from a fully observed event (e.g., AB, BC, AC) were statistically dependent, indicating a process of pattern completion, but retrievals from a partially observed event (e.g., AB, BC, CD) were not. However, inference for unseen “indirect” associations (i.e., AC, BD or AD) from a partially observed event showed strong dependency with each other and with linking direct associations from that event. In addition, inference of indirect associations correlated with the product of performance on the linking direct associations across events (e.g., AC with ABxBC) but not on the non-linking association (e.g., AC with CD). These results were seen across three experiments, with greater differences in dependency between indirect and direct associations when they were separately tested, but similar results following single and repeated presentations of the direct associations. The results could be accounted for by a simple auto-associative network model of hippocampal memory function. Our findings suggest that pattern completion supports recollection of fully observed multielement events and the inference of indirect associations in partly observed multielement events, mediated via the directly observed linking associations (although the direct associations themselves were retrieved independently). Together with previous work, our results suggest that associative inference plays a key role in reconstructive episodic memory and does so through hippocampal pattern completion.


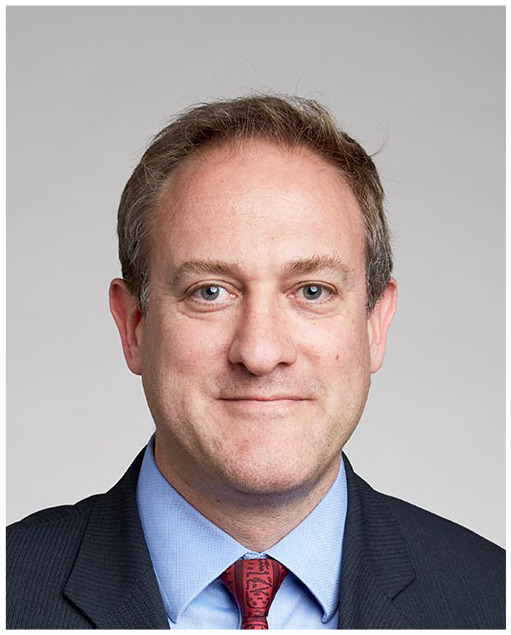


Neil Burgess, EPS mid-career prize winner

Episodic recollection is thought to reconstruct a coherent representation of a past event, incorporating existing knowledge and inferred information, rather than simply retrieving the remnants of veridical information stored during encoding (Bartlett, 1932; Eichenbaum, 2001; James, 1890; Schacter et al., 1998; Tolman, 1932; Tulving, 1985 Schacter & Addis, 2007a, 2007b). Episodic memories typically comprise numerous disparate elements from an experience that are bound together as a holistic representation (Davachi, 2006; Eichenbaum et al., 2007; Norman & O’Reilly, 2003; Tulving, 1985). However, not all aspects of a complex novel experience are necessarily attended or perceived during encoding, such as the individual associations between all possible pairs of elements comprising the event. Thus, during recall, when queried about such an association, one must generalise beyond what was attended or perceived to make novel inferences.

Here, we examined how episodic recollection can rebuild events inferred across overlapping novel associations and whether this could include previously unseen associations. We sought to determine whether the pattern of memory retrieval and level of inference across overlapping events can be explained by an auto-associative account of memory function. An important feature of associative binding in memory is that events are remembered in a holistic manner, supporting the recollective experience of retrieval that is a defining aspect of episodic memory (Tulving, 1985). Computational theories have long proposed that such holistic retrieval is supported by hippocampal pattern completion, with the presentation of a partial cue triggering reinstatement of all associated elements from an event (Gardner-Medwin, 1976; Marr, 1971; McClelland, 1995; Nakazawa et al., 2002; Wills et al., 2005).

Examining the pattern of associative retrieval across multielement events provides a useful tool in understanding how the associative structure of events contributes to memory performance. Previous studies have shown how retrievals of different paired associates from the same event are statistically related, suggesting that episodic memory reflects coherent representations supported by pattern completion (Horner et al., 2015; Horner & Burgess, 2013, 2014). In these studies, multimodal events involving a location, person, object and animal were encoded either with all elements simultaneously presented or with events built up over a series of overlapping pairwise associations. A subsequent memory test for all within event associations demonstrated that retrievals from the same event showed statistical dependency—the retrieval success of one association from an event was related to the retrieval success of other associations from the same event. Interestingly, when events were encoded as an open associative chain in which some but not all of the pairwise associations in the event were presented, the statistical dependency between retrievals from the same event was not observed (Horner et al., 2015; Horner & Burgess, 2014). That is, associative accuracy across multiple retrievals from an event showed a pattern consistent with independent storage of each of the overlapping associations. However, it is not known whether participants would be able to infer the unseen overlapping associations, if asked, and whether that would trigger pattern completion mechanisms apparently not used in retrieval of the observed associations.

Associative inference across learning episodes is often assessed using paradigms in which participants must recombine learned associations from overlapping experiences (e.g., AB, AC) to make judgements about indirect associations that were never experienced (e.g., BC; Carpenter & Schacter, 2017; Preston et al., 2004; Shohamy & Wagner, 2008; Zeithamova et al., 2016). Consistent with the role of the hippocampus in associative memory (Davachi, 2006; Eichenbaum, 2004), research has demonstrated its involvement in associative inference. For example, increases in hippocampal activity over the course of multiple encoding trials predict subsequent inference performance (Schlichting et al., 2014; Shohamy & Wagner, 2008; Zeithamova & Preston, 2010) and greater hippocampal activity accompanies successful retrieval of inferred associations (Heckers et al., 2004; Preston et al., 2004). Given the involvement of the hippocampus during these tasks, it is possible that associative inference relies on hippocampal pattern completion (Kuhl et al., 2010; Zeithamova et al., 2012). Studies assessing associative inference have also highlighted the importance of encoding repetition. Repeatedly learning overlapping events or associations can boost the ability to infer across them (Shohamy & Wagner, 2008; Zeithamova et al., 2012, 2016). In one study, fMRI repetition suppression was used to assess neural changes during presentation of overlapping pairs, each repeated three times (Zeithamova et al., 2016). Results demonstrated repetition suppression effects when non-overlapping pairs were repeatedly shown but increased activity in medial temporal lobe (MTL) structures during the presentation of overlapping pairs, even when these pairs were repeated multiple times. This increase in MTL activity correlated with associative inference performance suggesting that strengthened associations at encoding support increases in successful inference.

One question of interest is whether episodic recollection of partially observed events includes inference of the missing associations. Knowing that episodic memory is reconstructive in nature and malleable enough to merge directly observed associations with more general information (Bartlett, 1932; Eichenbaum, 2001; Eichenbaum et al., 1999; James, 1890; Tolman, 1932), we were keen to study the mechanisms underlying both the retrieval of observed associations and the inference of unseen associations, and how they might relate to each other.

We examined the relatedness (or “dependency”) of retrievals of both direct and inferred associations from the same multielement event, in events that were encoded across a series of overlapping pairwise associations (Horner et al., 2015; Horner & Burgess, 2014). For half of the events all associations between event elements were presented (AB, BC, AC; closed-loop structure), whereas the remaining events were encoded as an associative chain in which several possible associations were not presented (AB, BC, CD; open-loop structure). The presence of an additional fourth element in open-loop events was to ensure that both event structures had the same number of associations. We note that when open-loop events comprise the same number of elements as closed-loop events (but fewer associations, i.e., AB, AC versus AB, AC, BC), the absence of dependency for open-versus closed-loop events remains the same as when an open-loop event has four elements (Horner & Burgess, 2014). Extending previous studies, we tested memory for all observed pairs (direct associations) and all unobserved pairs (indirect associations or inferences, which were taken from open-loop events). In Experiment 1 we interleaved test trials for direct and indirect associations, following previous studies of memory inference. In Experiments 2 and 3, we sought to highlight the differences between memory for observed and inferred associations by separating the two types of test trials into two blocks. In Experiments 1 and 2, overlapping associations were experienced once each, similar to experiencing an ongoing situation in real life. In Experiment 3, we examined the effect of presenting overlapping associations only once or three times each, to investigate the effect of increasing the likelihood of successfully encoding direct associations. In Experiment 4, we present the performance of a simple associative memory model for comparison with our experimental results.

## Experiment 1—dependency across interleaved retrievals of direct and indirect associations within multielement events

### Method

#### Participants

Twenty-five healthy, English-speaking volunteers were recruited from the university student population. Data from all participants were used for memory performance analyses while data from 24 participants were used in dependency and performance correlation analyses (17 female, mean age = 26, age range: 22–36) after the exclusion of one participant due to performance exceeding 95% for direct pairs across all conditions. A power analysis based on effect sizes reported in previous studies (Horner & Burgess, 2014; η_*p*_^2^ range = .11–.48; *N* = 15) provided an estimate sample size needed for Experiment 1 (estimated *N* range = 9–24; power = 0.80, α = .05). Our proposed sample size of 25 corresponded to an estimate sample size of 24 based on an effect size of η_*p*_^2^ = .11 from earlier studies. The study was approved by the UCL Research Ethics Committee, and all participants gave informed written consent before taking part.

#### Materials

**Figure 1.**
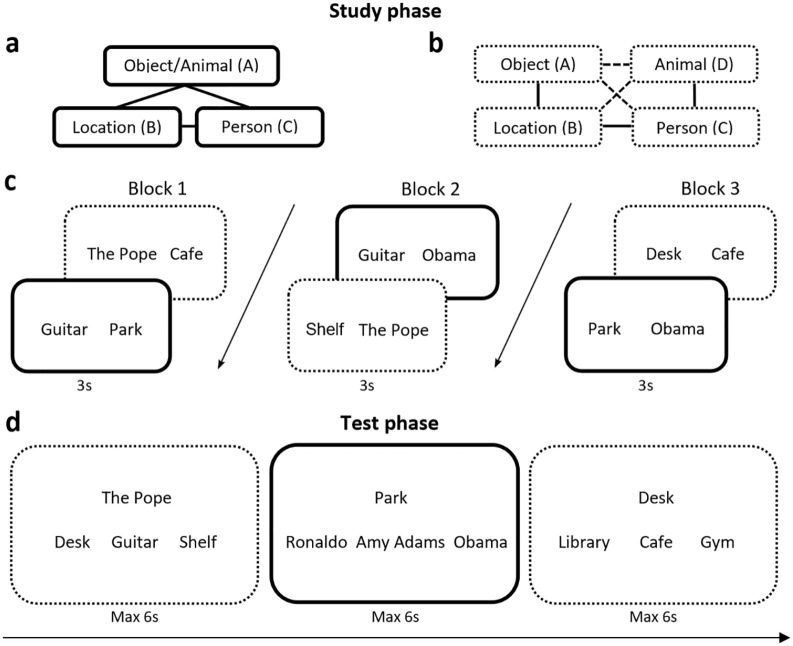
Design for Experiment 1. (a) Associative structure of a closed-loop A-B-C event. Half of the closed loops were location-person-object triads and the rest were location-person-animal triads. (b) Associative structure of an open-loop A-B-C-D event. Solid lines indicate trained, direct pairs while broken lines indicate indirect pairs inferred from trained pairs. (c) Study phase. Solid lines represent associations from closed-loop events while dotted lines represent associations from open-loop events. Line types are for illustration purposes only; type of event structure was not indicated in the study. (d) Test phase. Trials testing memory for indirect associations were presented before trials for direct associations from the same event, in a pseudorandomised order.

Stimuli included 60 locations, famous people, common objects and animals. For each participant, 60 novel events were created by randomly taking a location, person, object and animal for each event. Half of these events were assigned to the closed-loop A-B-C condition (location-person-object/animal respectively) (see Figure 1a) and the other half to the open-loop A-B-C-D condition (object-location-person-animal respectively) (see Figure 1b). As the closed-loop condition only used three elements within each event, half of the events were assigned to be location-person-object events and the other half location-person-animal events. For the open-loop condition, each event used all four elements (location-person-object-animal). Overall, this resulted in 30 closed- and 30 open-loop events.

#### Procedure

At encoding, events were presented as three separate, overlapping pairwise associations over three blocks (60 pairs per block; see Figure 1c). Presentation order within each block was randomised by individual pairs. For a trial, each pairwise associate was presented as text on screen for 3 s with participants instructed to imagine the two elements interacting in a meaningful way and as vividly as possible. Each encoding trial was preceded by a 0.5 s fixation cross and ended with a 0.5 s blank screen. For closed-loop events, participants saw all three overlapping pairwise associations from an event (e.g., AB, BC, AC), whereas open-loop events were encoded by omitting one association from the event structure (e.g., AB, BC, CD).

At retrieval, participants were tested on all direct associations (i.e., the pairs that they viewed at encoding) in each direction (e.g., cue with the location to retrieve the object, and cue with the object to retrieve the location) from all closed- and open-loop events, as well as all indirect associations (i.e., pairs inferred from those observed during encoding) in each direction from all open-loop events. This resulted in six associative memory trials for each event (360 test trials in total) and three indirect association trials for each open-loop event (180 test trials in total).

Presentation order was pseudorandom, mixing both direct and indirect trials but showing indirect trials from an event before the respective direct trials from that event, to prevent the earlier retrieval of direct associations assisting the later retrieval of indirect associations. Indirect associations had not been seen at study but could be inferred from encoded pairs through the underlying event structure (see Figure 1b). For example, while open-loop events were encoded over a series of trials as an associative chain (A-B-C-D), we could also test the three associations that were never shown but could be inferred by the participant (i.e., AC, BD, AD). Participants were told that for each test trial, the cue was linked to one of the presented options either directly or indirectly, and only one of the options was correct. This licenced them to make inferences in a way that might not occur in real life, an issue we return to in the General Discussion.

For each test trial, participants were presented with a fixation cross for 0.5 s followed by a text cue at the top centre of the screen, which could be a location, person, object, or animal (see Figure 1d). Three options were presented underneath the cue instead of six as in previous studies (Horner and Burgess, 2013; 2014; Horner et al., 2015) since pilot experiments generated low overall performance when six options were used in combination with the increase in the number of events, from 36 to the 60 used in this study. Participants were instructed to select, from these options, the correct associate paired either directly or indirectly with the cue via button press. On a single test trial, the three options were all previously seen items from the same category (e.g., three locations), and participants were given a total of 6 s to make a response.

#### Associative accuracy analysis

Associative accuracy scores were obtained for the closed- (A-B-C; object/animal-location-person respectively; see Figure 1a) and open-loop events (A-B-C-D; object-location-person-animal respectively; see Figure 1b). For direct associations, we collapsed performance across the six direct associations tested for each event (AB, BC, and AC for closed-loop events or CD for open-loop events, each pair in both directions). We then compared performance across closed- and open-loop conditions using paired samples *t*-tests. For indirect associations, we were interested in performance on each of the inferred pairs AC, BD, and AD independently. Therefore, we calculated associative accuracy for each indirect association separately, collapsing across testing direction. Performance was compared across pair-types using a one-way analysis of variance (ANOVA).

#### Dependency analysis

In accordance with previous studies (Horner et al., 2015; Horner & Burgess, 2013, 2014), we assessed dependency for direct associations within events by creating contingency tables for each participant for retrieving two elements from an event when cued by the other element from that event (A_B_A_C_ analyses where A is the common cue and B and C are the targets) and for retrieving one element when cued by the other two elements (B_A_C_A_ analyses where A is the common target). This measure therefore reflects how retrieving one association from an event depends on the retrieval of another association from the same event (see Table 1). For each participant, we constructed four separate contingency tables for each of the experimental conditions (closed- and open-loop events). Thus, tables were created for (1) cueing with the location—the location A_B_A_C_ analysis; (2) retrieving the location—the location B_A_C_A_ analysis; (3) cueing with the person—person A_B_A_C_ analysis; and (4) retrieving the person—person B_A_C_A_ analysis. For evaluating dependency of direct associations on other within-event direct associations across open-loop and closed-loop events, only pairs with person or place as the common cue or target were used (as objects and animals were not present in all events). However, this restriction did not apply for other dependency analyses involving indirect associations, since only open-loop events were studied and all of them had the same location-person-object-animal structure.

**Table 1.** Contingency table for the Independent model, presenting the frequency (over events) of the four combinations of correct or incorrect retrieval of elements B and C when cued by A.

Retrieval of Element C	Retrieval of Element B	
	Correct (*P*_AB_)	Incorrect (1 – *P*_AB_)
Independent Model		
Correct (*P*_AC_)	$\sum_{i=1}^{N}P_{\mathrm{AB}}P_{\mathrm{AC}}$	$\sum_{i=1}^{N}P_{\mathrm{AC}}\left(1-P_{AB}\right)$
Incorrect (1—*P*_AC_)	$\sum_{i=1}^{N}P_{\mathrm{AB}}\left(1-P_{\mathrm{AC}}\right)$	$\sum_{i=1}^{N}\left(1-P_{\mathrm{AB}}\right)\left(1-P_{\mathrm{AC}}\right)$

In the Independent model, the probability of correctly retrieving B when cued by A (across all events) is *P*_AB_.

Data from each contingency table were compared to those predicted for each participant by the Independent model of retrieval (see Table 1). The Independent model estimates the level of dependency expected if all retrievals within an event are independent and so controls for any effects of overall performance. Participants with over 95% accuracy for direct associations across all conditions were removed from dependency analyses since high performance prevents detection of any differences from the Independent model.

Overall, we calculated dependency for each condition based on the proportion of events where both associations were either correctly or incorrectly retrieved. Dependency values from our data ranged from 0.5 (*full independence*) to 1 (*full dependence*).

As dependency scales with accuracy, only comparisons between the data and Independent model are meaningful. The difference between the two (Ddata-Di) hence acted as our measure of dependency in a condition; a condition could be said to exhibit dependency if dependency in the data (Ddata) was significantly more than the value estimated by the Independent model (Di).

The Independent model gives the level of dependency expected for that participant’s performance levels on the associations in question, assuming that they are remembered independently. The data can exhibit less dependency than predicted by the Independent model (i.e., Ddata < Di), as might arise due to interference or competition between associations from the same event, such that the successful retrieval of AB hinders the retrieval of AC.

To examine the dependency across retrievals and the ability to infer associations, we next calculated dependency for the unseen indirect pairs from the open-loop events. This dependency was compared with that among direct associations from the same event (in this case covering all pairs of direct associations, not just those with location or person as the common cue or target).

We also derived the dependency of indirect pairs on linking direct pairs and the dependency of indirect pairs on non-linking direct pairs. Linking associations refer to the direct pairs on pathways creating the indirect pair. For example, if the indirect pair is AC, the linking pairs are AB and BC. Non-linking associations are directly observed pairs from the same event that were not on the pathway potentially supporting the inference; for instance, CD is non-linking for indirect association AC.

Dependency of indirect pairs on linking pairs would indicate that the inferred association depends on the strength of the direct linking associations, as would be the case for a pattern completion explanation of inference. Under a pattern completion explanation, inference of AC would be possible via the spreading of activity from A to C via the learned direct associations AB and BC. Dependency of indirect pairs on non-linking pairs was analysed for comparison with their dependency on linking associations. For inference of AD, all observed pairs (AB, BC, and CD) are linking pairs.

To establish dependency, after log transformation (Equation 1), one-sample *t*-tests on Ddata-Di in each condition were conducted, and ANOVAs performed to study dependency also used Ddata-Di as a measure of dependency

(1) $${(Ddata-Di)}_{i}\prime =\log[{\left(Ddata-Di\right)}_{i}+1]$$

Shapiro-Wilk tests were used to measure normality of data distribution. Ddata-Di values in all dependency analyses were log-transformed (Equation 1) due to the non-Gaussian distributions of dependency across direct pairs for closed events, *W*(24) = .819, *p* = .001, and that for open events, *W*(24) = .732, *p* < .001, dependency of indirect pairs on direct linking pairs, *W*(24) = .776, *p* < .001, and dependency of AD on all linking pairs, *W*(24) = .776, *p* < .001.

As an alternative measure of whether inference of an indirect pair relates to the spreading of activity through direct linking associations, we correlated participants’ accuracy scores for indirect associations with the product of their accuracy scores for direct linking associations, across events. Correlation between performance on indirect pairs and on the corresponding non-linking direct associations was also performed for comparison. For example, we computed the Pearson correlation coefficient between accuracy scores for indirect association AC and the product of accuracy scores for direct associations AB and BC on the same trial. Note that the accuracy score for a given association is 0, 1, or 2, because each association was tested twice (in either direction). The *r* values for each participant were then subjected to a Fisher’s *Z*-transformation followed by a one-sample *t*-test to determine if there was a correlation between retrieval accuracy for indirect associations and retrieval accuracy for the respective linking and non-linking associations across participants. Participants who got all of the relevant associations in a single condition correct or all incorrect were removed from this analysis, causing sample size *N* to vary.

### Results

#### Associative accuracy

**Figure 2.**
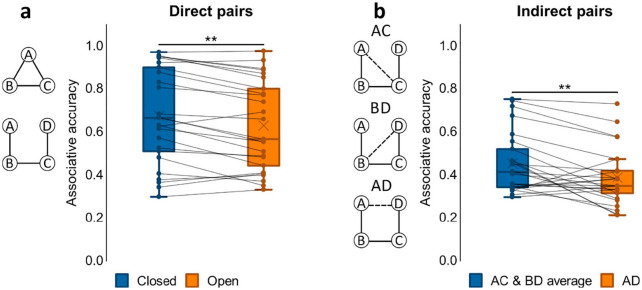
Associative accuracy results for Experiment 1. (a) Proportion correct retrievals for direct pairs in closed and open loops. (b) Proportion correct retrievals overall in indirect pairs AD, BD and AD. **p < .01. *N* = 25 for both a and b.

We first examined associative accuracy for direct associations across closed- and open-loop events. A paired samples *t*-test on associative memory performance showed greater accuracy for closed- compared to open-loop events, *t*(24) = 3.74, *p* = .001, *d* = .748; see Figure 2a. As only open-loop events included indirect associations at test, we separately analysed open-loop events, comparing average accuracy for direct with indirect associations, i.e., pairs not observed at encoding. We found a significant difference between conditions with greater performance for direct associations, *t*(24) = 7.67, *p* < .001, *d* = 1.53. Nonetheless, a one-sample *t*-test on accuracy for indirect associations showed that mean performance was greater than chance, *t*(24) = 3.89, *p* < .001, *d* = .779; chance = 0.33.

To further assess performance on indirect associations we next performed a one-way ANOVA on accuracy for the different indirect pair-types (AC, BD, AD; see Figure 2b). We found a significant effect of pair-type, *F*(2, 48) = 6.10, *p* = .004, η_*p*_^2^ = .203, that was due to worse accuracy for the AD pair when compared to both AC, *t*(24) = 3.58, *p* = .001, *d* = .717, and BD, *t*(24) = 2.33, *p* = .029, *d* = .465. Since AC and BD were each inferred across two direct associations (AB and BC for AC; BC and CD for BD) while inference of AD involved a chain of three direct associations (AB, BC, and CD), it made sense that performance for AC and BD was comparable and performance for AD was worse than their average.

#### Dependency across direct associations

**Figure 3.**
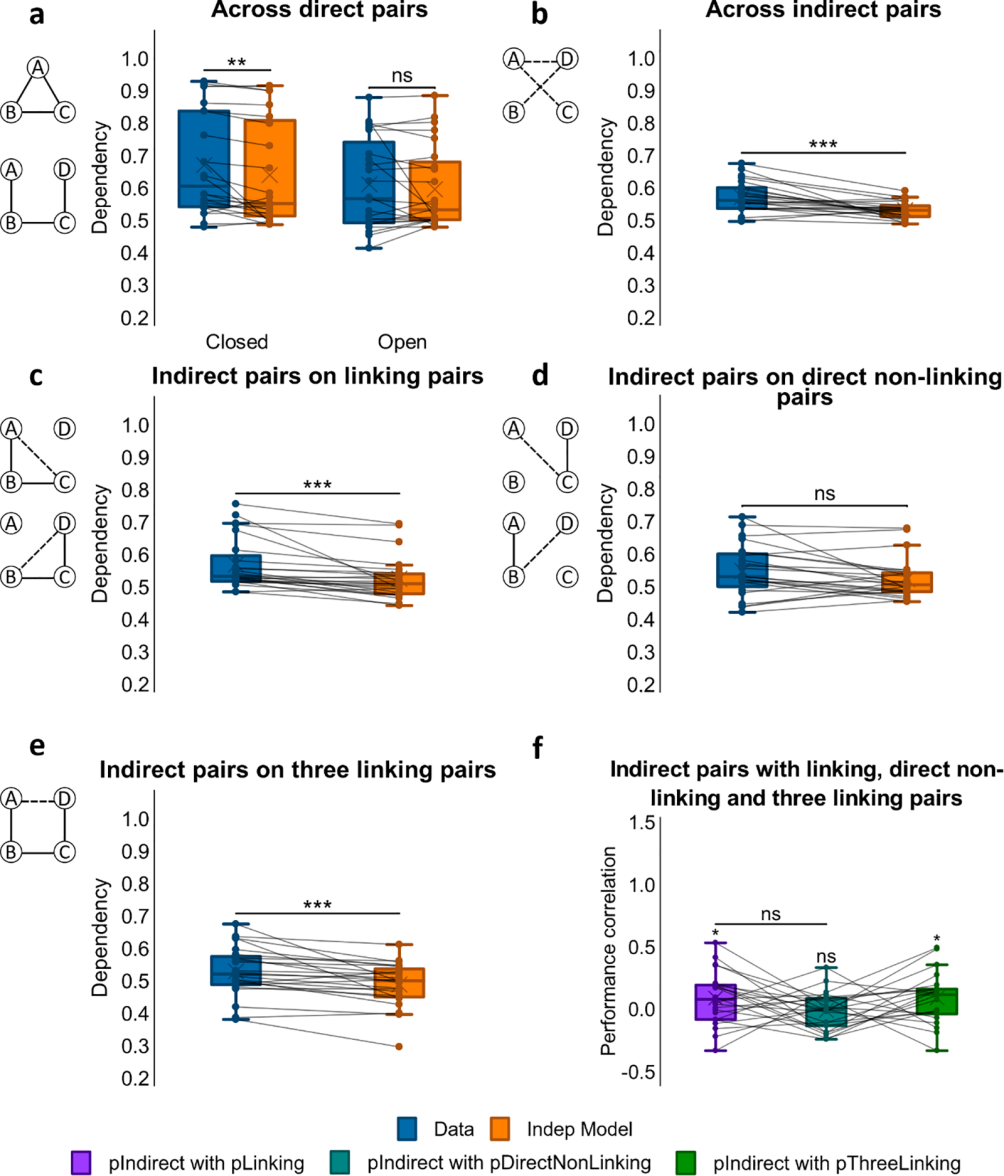
Dependency results for Experiment 1. (**a**) Dependency of direct pairs on other direct pairs from the same event for closed and open loops, and corresponding Independent model. (**b**) Dependency of indirect pairs on other indirect pairs from the same event for open loops, and corresponding Independent model. (**c**) Dependency of indirect pairs on all related direct pairs from the same event for open loops, and corresponding Independent model. (**d**) Dependency of indirect pairs on unrelated direct pairs from the same event for open loops, and corresponding Independent model. (**e**) Dependency of indirect pair AD on all direct linking pairs from the same event for open loops, and corresponding Independent model. (**f**) Performance correlation between indirect associations and direct linking, direct non-linking (for AC and BD) and three linking direct associations (for AD; * within a column reflects a significant difference from zero). ^***^p < .001; ^**^p < .01; *p < .05; ns not significant. *N* = 24 for **a**-**f**.

Dependency was first assessed for the direct associations that had been presented at encoding, looking for differences between closed- and open-loop events (see Figure 3a). Analysis using a one-way ANOVA showed no significant difference in dependency between closed- and open-loop structures, *F*(1, 23) = 2.79, *p* = .108, but one-sample *t*-tests showed significant dependency in closed-loop events, *t*(23) = 3.08, *p* = .005, *d* = .628, which was absent in open-loop events, *t*(23) = 0.772, *p* = .448.

#### Dependency across indirect associations

We next examined dependency across all indirect associations from open-loop events (AC,(AC, BD, AD) using a one-sample *t*-test on dependency (see Figure 3b). Analysis demonstrated retrieval dependency among indirect associations, *t*(23) = 4.12, (*p* < .001, *d* = .842). A paired samples *t*-test was also performed on dependency across direct and indirect pairs but no significant difference was found, *t*(23) = –1.59, *p* = .126.

#### Dependency of indirect associations on direct linking associations

To examine relatedness across indirect and direct associations, we analysed the amount of dependency in retrievals of indirect associations on direct associations that would be required to make an inference. For example, successfully retrieving the indirect association AC would be expected to rely on the retrieval of direct associations AB and BC, and the successful retrieval of the indirect association BD on the retrieval of direct associations BC and CD (see Figure 3c). A one-sample *t*-test showed dependency, *t*(23) = 4.32, *p* < .001, *d* = .882, suggesting that the ability to infer indirect associations was related to the retrieval success of related overlapping direct associations.

To further assess if the retrieval of indirect associations (AC, BD) was related to the probability of retrieving both of the corresponding linking direct associations (e.g., AB and BC for AC), we correlated performance scores across events for each participant (see Figure 3f). Specifically, we computed a Pearson correlation coefficient between accuracy scores for the indirect associations with the product of accuracy scores for the two linking direct associations. A one-sample *t*-test indicated that Fisher’s *Z*-transformed correlation coefficients across participants were significantly greater than zero, mean *r* = .095, *t*(22) = 2.30, *p* = .031, *d* = .480, suggesting that the retrieval of inferred associations was related to the retrieval of their linking pairs across events.

#### Dependency of indirect associations on direct non-linking associations

We next looked at the dependency of retrieving indirect associations on the retrieval success of direct unrelated associations, which would not be required to make an inference. That is, we assessed dependency for retrieving the indirect association AC on the retrieval of the direct association CD, and also dependency for retrieving the indirect association BD on the retrieval of the direct association AB (see Figure 3d). In this case, a one-sample *t*-test showed no significant dependency, *t*(23) = 1.85, *p* = .077.

Dependency of indirect associations on direct non-linking pairs was also compared with their dependency on direct linking pairs. A paired sample *t*-test on the two revealed greater dependency for inferred associations on direct linking pairs than on direct non-linking pairs, *t*(23) = 2.08, *p* = .049.

The correlation between performance on inferred associations (AC, BD) and on their direct non-linking pairs (CD, AB respectively) across events for each participant was also examined (see Figure 3f). However, Fisher Z-transformed Pearson correlation coefficients (*r*) across participants were not significantly different from zero, mean *r* = −.0004, *t*(22) = 0.022, *p* = .982. Therefore, we found no evidence for a relationship between accuracy for inferred associations and accuracy for non-linking associations across open-loop events.

#### Dependency of indirect association AD on all linking direct associations

We next examined the dependency of retrieving the indirect association AD on all direct associations that would be required to form an associative chain supporting the correct inference, i.e., AB-BC-CD (see Figure 3e). A one-sample *t*-test reported significant dependency, *t*(23) = 3.88, *p* < .001, *d* = .793.

To further examine whether the retrieval success of AD across events was related to the successful retrieval of the whole associative chain of AB, BC, and CD, we correlated accuracy scores for AD with the product of accuracy scores for AB, BC, and CD (see Figure 3f). Fisher’s Z-transformed *r* values across participants were significantly greater than zero, mean *r* = .098; *t*(21) = 2.34, *p* = .029, *d* = .499, indicating that performance on AD was correlated to performance on the entire chain of AB, BC, and CD associations across events.

### Summary of Experiment 1

In accordance with previous research, Experiment 1 demonstrated dependency in the retrieval of direct pairwise associations from events encoded as closed- compared to open-loop structures. For open-loop events, performance across indirect associations from the same event (e.g., AC, BD, AD) also showed statistical dependency. Performance in inferring pairwise associations (e.g., AC) was statistically dependent on retrieval of the linking direct associations (e.g., AB, BC), but not on retrieval of the unrelated direct associations (e.g., CD). In addition, performance on inferred associations correlated, across events, with the product of retrieval performance on the linking direct associations.

Altogether, these results tentatively point to an auto-associative network in which all associations are stored as a linked network to support holistic retrieval, consistent with a role of pattern completion. The presence of dependency among indirect associations, but not direct associations, in open-loop events was likely because direct associations could be remembered independently, each from the observation of their own presentation, whereas inferential judgements were contingent on within-event pattern completion.

## Experiment 2—dependency across separate retrievals of direct and indirect associations within multielement events

Experiment 1 followed previous studies on inference, using interleaved test trials on directly observed and indirect (inferred) associations (Banino et al., 2016; Preston et al., 2004; Schlichting et al., 2014; Shohamy & Wagner, 2008; Zeithamova & Preston, 2010) to test both types of association where participants had no expectation of encountering one over the other. In addition, inferential trials were presented before trials testing direct associations from the same event to prevent the latter from aiding in the retrieval of the former. However, as we were interested in the differences between answers to questions about indirect associations (“inference”) and directly observed associations (“memory”) indicated in Experiment 1, we sought to explicitly maximise any difference in processing between the two types of test and so make each easier to study, in isolation from the other. Thus, in Experiment 2, the different types of test were separated into two sessions, with the trials for direct associations in one session followed by trials for indirect associations in the next.

### Method

#### Participants

Thirty-four healthy, English-speaking volunteers from the university student population gave informed consent to participate. All participants were included in memory accuracy analyses but only 33 were included in dependency and performance correlation analyses (24 female, mean age = 24, age range: 18–33) after removing one participant who scored above 95% accuracy for direct pairs across all conditions. An approximate sample size needed for Experiment 2 (estimated *N* range = 8–47; power = 0.80, α = .05) was obtained from a power analysis based on effect sizes reported in Experiment 1 (η_*p*_^2^ range = .15–.41, *N* range = 24–25). As the median estimated sample size within the approximated range is 28, our sample size of 34 would be more than adequate for the main objective of this study.

#### Materials

The stimuli used were the same as those in Experiment 1 (see Figure 1a and b).

#### Procedure

The study procedure was similar to that in Experiment 1 (see Figure 1c and d), except that the test phase was split into two consecutive sessions. The first consisted of trials testing direct associations (see Figure 1a) and the second consisted of trials testing indirect associations (see Figure 1b). Participants were only informed at the start of the second test session that their memory would be tested on the associations that had not been seen at study but could be inferred from encoded pairs via the underlying event structure. As a result, inferences had to be actively made, although in the real world they would not necessarily be called for (see General Discussion). In both sessions, participants were instructed to select the correct paired associate out of three options for the cue shown on screen during each trial. Presentation order within each session was randomised.

#### Associative accuracy analysis

Associative memory performance was analysed as in Experiment 1.

#### Dependency analysis

Dependency was analysed as in Experiment 1. Log transformation (Equation 1) was applied to all Ddata-Di analyses after a Shapiro-Wilk test of normality reported that dependency across direct pairs over both types of loops, *W*(24) = .624, *p* < .001, and dependency across indirect pairs, *W*(33) = .916, *p* = .014, had a non-Gaussian distribution.

### Results

#### Associative accuracy

**Figure 4.**
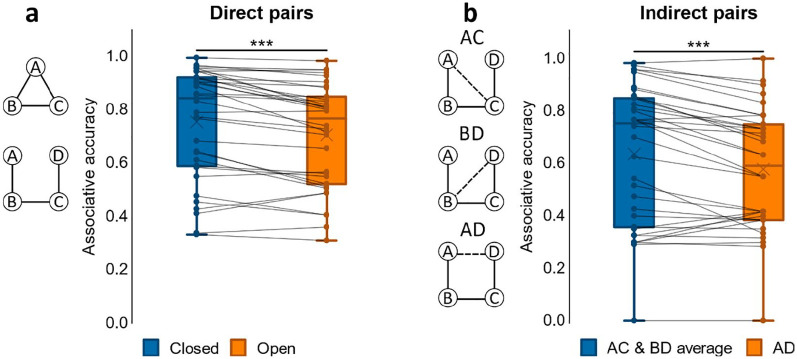
Associative accuracy results for Experiment 2. (a) Proportion of correct retrievals for direct pairs in closed and open loops. (b) Proportion of correct retrievals overall in indirect pairs AD, BD, and AD for open loop events. ***p < .001. *N* = 34 for both a and b.

As in Experiment 1, associative accuracy for direct associations was compared between closed- and open-loop events using a paired samples *t*-test, which showed greater accuracy for closed- compared to open-loop events, *t*(33) = 4.79, *p* < .001, *d* = .821, see Figure 4a. We then analysed indirect associations by comparing average accuracy with that for direct associations in open-loop events, since indirect associations were derived from open-loop events only. Average accuracy for direct associations was higher than it was for indirect associations, *t*(33) = 4.27, *p* < .001, *d* = .732. Nonetheless, a one-sample *t*-test on accuracy for indirect associations revealed that mean performance was greater than chance, *t*(33) = 6.62, *p* < .001, *d* = 1.14; chance = 0.33.

Next, we examined accuracy for indirect associations further by conducting a one-way ANOVA on accuracy for the different indirect pair-types (AC, BD, AD; see Figure 4b). There was a significant effect of pair-type, *F*(2, 66) = 11.4, *p* < .001, η_*p*_^2^ = .256, as accuracy for the AD pair was worse than that for both AC, *t*(33) = 4.10, *p* < .001, *d* = .704, and BD, *t*(33) = 3.42, *p* = .002, *d* = .587.

#### Dependency across direct associations

**Figure 5.**
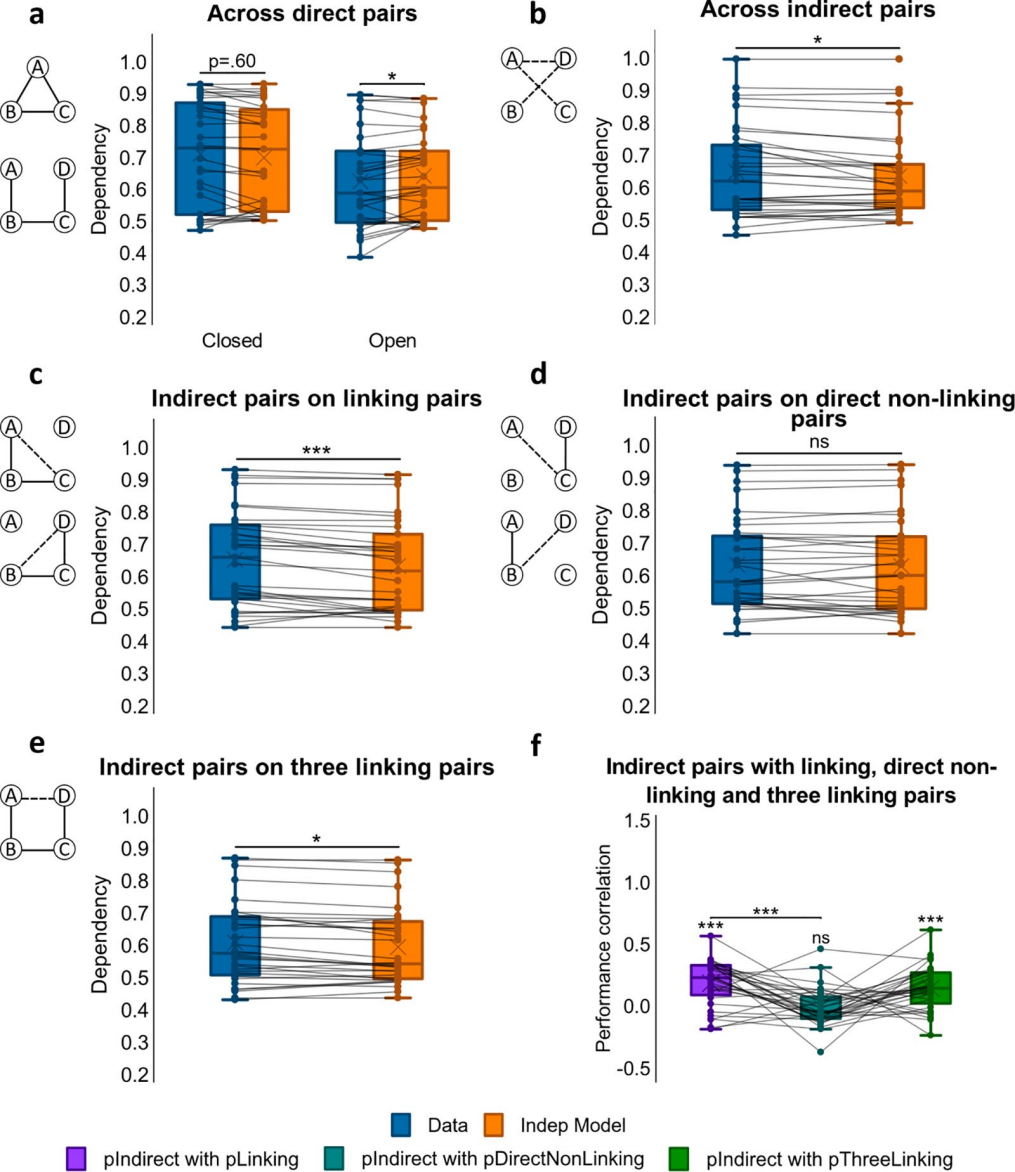
Dependency results for Experiment 2. (**a**) Dependency of direct pairs on other direct pairs from the same event for closed and open loops, and corresponding Independent model. (**b**) Dependency of indirect pairs on other indirect pairs from the same event for open loops, and corresponding Independent model. (**c**) Dependency of indirect pairs on all related direct pairs from the same event for open loops, and corresponding Independent model. (**d**) Dependency of indirect pairs on all unrelated direct pairs from the same event for open loops, and corresponding Independent model (**e**) Dependency of indirect pair AD on all direct linking pairs from the same event for open loops, and corresponding Independent model. (**f**) Performance correlation between indirect associations and direct linking, direct non-linking (for AC and BD) and three linking direct associations (for AD; *** within a column reflects a significant difference from zero). ^***^p < .001; *p < .05; ns not significant. *N* = 33 for **a**-**f**.

To examine the dependency of direct associations on other within-event direct associations, we compared the level of dependency across closed- and open-loop events (see Figure 5a) as in Experiment 1. A one-way ANOVA demonstrated significant difference in dependency, *F*(1.45, 36.3) = 16.8, *p* < .001, η_*p*_^2^ = .402, with greater dependency observed in closed- compared to open-loop events, *t*(32) = 3.41, *p* = .002 *d* = .593. Analysing the findings further using one-sample *t*-tests, we saw a tendency for dependency in closed-loop events, *t*(32) = 1.87, *p* = .070, *d* = .326, whereas in open-loop events, dependency was lower than predicted by the Independent model, *t*(32) = −2.63, *p* = .013, *d* = −.458, suggesting interference or retrieval induced forgetting between within-event associations, such that the successful retrieval of one impairs retrieval of others from the same event.

#### Dependency across indirect associations

We next assessed dependency across all indirect associations from open-loop events (AC, BD, AD) using a one-sample *t*-test (see Figure 5b), which revealed significant dependency, *t*(32) = 2.10, *p* = .043, *d* = .366. A paired samples *t*-test was performed on the dependency across direct associations and across indirect associations, revealing that dependency was higher among indirect associations than among direct associations in open loops, *t*(32) = −3.11, *p* = .004, *d* = −.542.

#### Dependency of indirect associations on linking direct associations

Next, we assessed how related indirect and direct associations were by measuring the dependency of retrieving indirect associations on retrieving the direct associations that linked their constituent elements. Specifically, the retrieval of AC would likely be dependent on the retrievals of AB and BC, and the retrieval of BD would likely be dependent on the retrievals of BC and CD (see Figure 5c). A one-sample *t*-test reported dependency, *t*(32) = 3.86, *p* < .001, *d* = .671, indicating that the ability to infer indirect associations was related to the successful retrieval of overlapping direct associations.

To further examine if retrieval of indirect associations was related to the successful retrieval of linking associations across events, we correlated participants’ performance in indirect associations with the product of performance in the direct linking pairs (see Figure 5f) and conducted a Fisher’s Z**-**transformation of the Pearson *r* values. A one-sample *t*-test indicated that transformed *r* values across participants were significantly greater than zero, mean *r* = .189; *t*(31) = 6.12, *p* < .001, *d* = 1.08. Retrieval of inferred associations was thus correlated with the successful retrieval of their linking direct associations across events.

#### Dependency of indirect associations on direct non-linking associations

Retrieving indirect associations was not expected to rely on successfully retrieving unrelated direct associations—that is, the retrieval of AC seems less likely to depend on the successful retrieval of CD, and the retrieval of BD on the successful retrieval of AB (see Figure 5d). As expected, a one-sample *t*-test showed that there was no significant dependency, *t*(32) = 0.601, *p* = .552.

We also looked at how dependent indirect associations were on direct linking associations and on direct non-linking associations. A paired samples *t*-test demonstrated that the dependency of inferred associations on direct linking associations was significantly higher than their dependency on direct non-linking associations, *t*(32) = 2.13, *p* = .041, *d* = .371.

We then looked at whether dependency between retrieval of indirect associations (AC, BD) and retrieval of their direct non-linking associations held true across events (CD, AB respectively; see Figure 5f). For every participant, accuracy scores of the inferred associations across events were correlated with the product of the accuracy scores of the respective non-linking pairs. In this case, Fisher’s *Z*-transformed *r* values across participants were not significantly different from zero, mean *r* = .005; *t*(31) = 0.209, *p* = .836, suggesting that across open-loop events, the successful retrieval of non-linking associations was not pertinent to that of inferred associations.

#### Dependency of indirect association AD on all direct associations

We then measured the dependency of retrieving the indirect association AD on all direct associations necessary to make the correct inference, i.e., AB-BC-CD (see Figure 5e). Performing a one-sample *t*-test, we found significant dependency, *t*(32) = 2.59, *p* = .014, *d* = .450. To further examine the relatedness between retrieval of AD and retrieval of the whole linking chain AB-BC-CD across open-loop events, participants’ accuracy scores for AD were correlated with the product of the accuracy scores for AB, BC and CD (see Figure 5f). Fisher’s Z-transformed correlation coefficients were significantly higher than zero, mean *r* = .149, *t*(31) = 4.92, *p* < .001, *d* = .870, indicating that retrieval of the inferred AD association was associated with retrieval of the direct associations comprising the AB-BC-CD chain across open-loop events.

### Summary of Experiment 2

Despite differences in testing procedure—Experiment 1 testing direct and indirect associations alternately with indirect associations shown before direct associations from the same event, and Experiment 2 testing them separately, with direct associations first—both experiments revealed dependency among inferred associations within an event. While average performance was higher in Experiment 2, dependency results remained relatively similar. As opposed to Experiment 1, greater dependency was shown for the retrieval of direct associations from events with closed-loop structures than for events with open-loop structures. Even so, unlike the case in Experiment 1, inferential judgements across indirect associations from the same open-loop event were statistically dependent for retrieval perhaps because all of them relied on the direct linking association BC. Associative inference was also statistically dependent on retrieval of the linking direct associations, but not the irrelevant direct associations. As in Experiment 1, we found a significant correlation between performance on inferring indirect associations and the product of performances on retrieving the linking direct associations across events.

These observations reinforce the view that an auto-associative network supports the retrieval of both learned and inferred associations from a multielement event in an integrated manner. Inferences made across directly learned associations reflected pattern completion between indirect and direct linking associations, and such dependency was stronger in Experiment 2, where participants knew which questions referred to direct associations and which to indirect associations, than in Experiment 1. This difference in dependency was perhaps due to the testing of direct associations preceding that of indirect associations in Experiment 2, providing the opportunity to strengthen encoded pairs before making inferences that rely on them. Such practice was not afforded in Experiment 1 where testing was alternating and indirect associations from an event were tested before its respective direct associations, though within-event dependency was still evident.

## Experiment 3—effects of repeated presentation on inference and dependency of within-event associations

The results of Experiment 2 emphasise the difference in processing between indirect and direct associations—showing greater dependency among indirect associations, even though performance was weaker. In Experiment 3, we attempted to manipulate the probability of successfully encoding direct associations by presenting them either once or repeated three times during the study phase. Previous research has shown that repetition of overlapping associations improves memory for associations inferred across them (Zeithamova et al., 2016). We thus sought to study the effect of repetition on the dependency of direct pairs in open-loop events. The two experiments also differed in terms of the number of foil items presented in each test trial, which was increased from three to six to better replicate earlier studies, given the anticipated improvement in performance with repeated presentations (Horner et al., 2015; Horner & Burgess, 2013, 2014).

### Method

#### Participants

Forty-three healthy volunteers were enlisted from the university student population. Data from all participants were used to analyse memory performance, but data from only 42 participants were used to analyse dependency and performance correlation (28 female, ages 19–35, mean age = 24, 3 left-handed) after one exclusion due to performance exceeding 95% in direct associations across all conditions. A power analysis on effect sizes noted in Experiment 2 (η_*p*_^2^ range = .12–.49, *N* range = 33–34) provided an approximate sample size for Experiment 3 (estimated *N* range = 9–99; power = 0.80, α = .05). Seeing that an effect size of .29 from Experiment 2 estimated a sample size of 35, we studied a sample size of 43 which should allow for further subgroup analyses, since Experiment 3 additionally examined repetition effects.

#### Materials

The stimuli used were similar to those in Experiments 1 and 2 (see Figure 1a and b) with the following exceptions. Thirty multimodal events were generated for each closed- and open-loop condition, but 60% of events within each condition were repeated and 40% were not. We chose to repeat a larger proportion of events given that an earlier pilot experiment in which the split was equal produced accuracy rates for the repeated condition that were too high for the dependency model to process. Overall, this resulted in 12 closed-loop single presentation (Single Closed condition), 18 closed-loop repeated presentation (Repeated Closed), 12 open-loop single presentation (Single Open), and 18 open-loop repeated presentation events (Repeated Open).

#### Procedure

The study procedure was the same as that in Experiments 1 and 2 (see Figure 1c and d), except that it involved repetition during encoding—encoding trials were presented across three sessions, each of which comprised three blocks—and there were now six options at test instead of three. Events in the repeated presentation condition were repeated three times, and the order of pairwise associations within a block random (e.g., AB, BC, AC in block 1; AC, BC, AB in block 2; BC, AC, AB in block 3), while events in the single presentation condition were shown once. Within each session, all three pairwise associations from events assigned to the repeated presentation condition (18 closed-loop and 18 open-loop) were shown, with one pairwise association in each block. One pairwise association from events assigned to the single presentation conditions (12 closed-loop and 12 open-loop) was also shown in each session. For the second and third sessions, all associations from repeated presentation events were again shown across blocks and the second and third pairwise associations from single presentation events were shown across Blocks 2 and 3, respectively (giving 396 encoding trials in total). The order of encoding different pair-types was randomised within each block. The procedure for testing followed that in Experiment 2.

#### Associative accuracy analysis

Associative accuracy scores were analysed in the same way as in Experiments 1 and 2, with the addition of calculating average scores separately for repeated and single conditions. Repeated measures ANOVAs were also performed, to study the effects of loop-type, encoding repetition and type of association.

#### Dependency analysis

Dependency was calculated as in Experiments 1 and 2, but we also examined differences between dependency in the data (Ddata) and Independent (Di) model for single and repeated loops separately. A Shapiro-Wilk test of normality revealed a deviation from normality for dependency across direct pairs in repeated events, *W*(42) = .940, *p* = .028; closed events, *W*(42) = .939, *p* = .026; single closed events, *W*(42) = .929, *p* = .012; and repeated closed events, *W*(42) = .944, *p* = .040, as well as dependency across indirect pairs in single open events, *W*(42) = .929, *p* = .012, repeated open events, *W*(42) = .912, *p* = .003, and overall across repetition, *W*(42) = .895, *p* = .001, and dependency of indirect pairs on direct linking pairs in repeated open events, *W*(42) = .879, *p* < .001. In light of these findings, a log transformation (Equation 1) was implemented in all Ddata-Di analyses.

### Results

#### Associative accuracy

**Figure 6.**
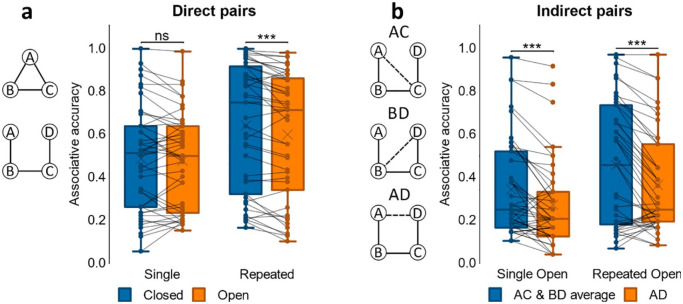
Associative accuracy results for Experiment 3. (a) Proportion correct retrievals in Single and Repeated events for direct pairs in closed and open loops. (**b**) Proportion correct retrievals in Single and Repeated events for indirect pairs AC, BD, and AD. ***p < .001; ns not significant. *N* = 43 for both a and b.

We first assessed associative accuracy (see Figure 6a) for direct associations (i.e., pairs that had been shown at encoding) by looking across closed- and open-loop events that had been repeated or not at encoding. Analysis using a 2x2 ANOVA (loop-type, repetition) showed an interaction between loop-type and repetition with a trend towards significance, *F*(1, 42) = 4.05, *p* = .051, η_*p*_^2^ = .088. We also saw a significant main effect of loop-type, *F*(1, 42) = 12.7, *p* < .001, η_*p*_^2^ = .232, due to better memory performance for closed- compared to open-loop events, and a main effect of repetition, *F*(1, 42) = 63.3, *p* < .001, η_*p*_^2^ = .601, with better performance for those associations that had been repeated versus associations seen only once at encoding.

Similar to Experiments 1 and 2, as closed-loop structures did not include indirect associations, we next separately examined open-loop structures and compared associative accuracy between direct and indirect associations using a 2x2 ANOVA (association-type x repetition). Results from this analysis showed no significant interaction, *F*(1, 42) = 0.812, *p* = .373, but we did observe significant main effects of both association type, *F*(1, 42) = 74.2, *p* < .001, η_*p*_^2^ = .639, and repetition, *F*(1, 42) = 42.01, *p* < .001, η_*p*_^2^ = .500, due to better memory performance for direct associations and for repeated events.

To further assess performance on indirect associations from open-loop structures, we next looked at accuracy across each of the different pair-types that had not been explicitly paired at encoding (i.e., indirect pairs AC, BD, AD; see Figure 6b). Using a 3x2 ANOVA (pair-type, repetition), we found a significant main effect of pair-type, *F*(1.78, 74.6) = 14.7, *p* < .001, η_*p*_^2^ = .259, due to better associative accuracy for AC, *t*(42) = 5.15, *p* < .001, *d* = .785, and BD pairs, *t*(42) = 4.40, *p* < .001, *d* = .671, compared to AD, with no difference in accuracy between AC and BD, *t*(42) = −.860, *p* = .395. We also found a main effect of repetition, *F*(1, 42) = 24.8, *p* < .001, η_*p*_^2^ = .371, due to better performance for repeated pairs, but no pair-type x repetition interaction, *F*(2, 84) = 1.56, *p* = .216. Taken together, these results indicate that repetition improved performance on both direct and indirect associations, and in terms of accuracy, indirect pairs AC and BD were comparable and each retrieved more accurately than the indirect pair AD.

#### Dependency across direct associations

**Figure 7.**
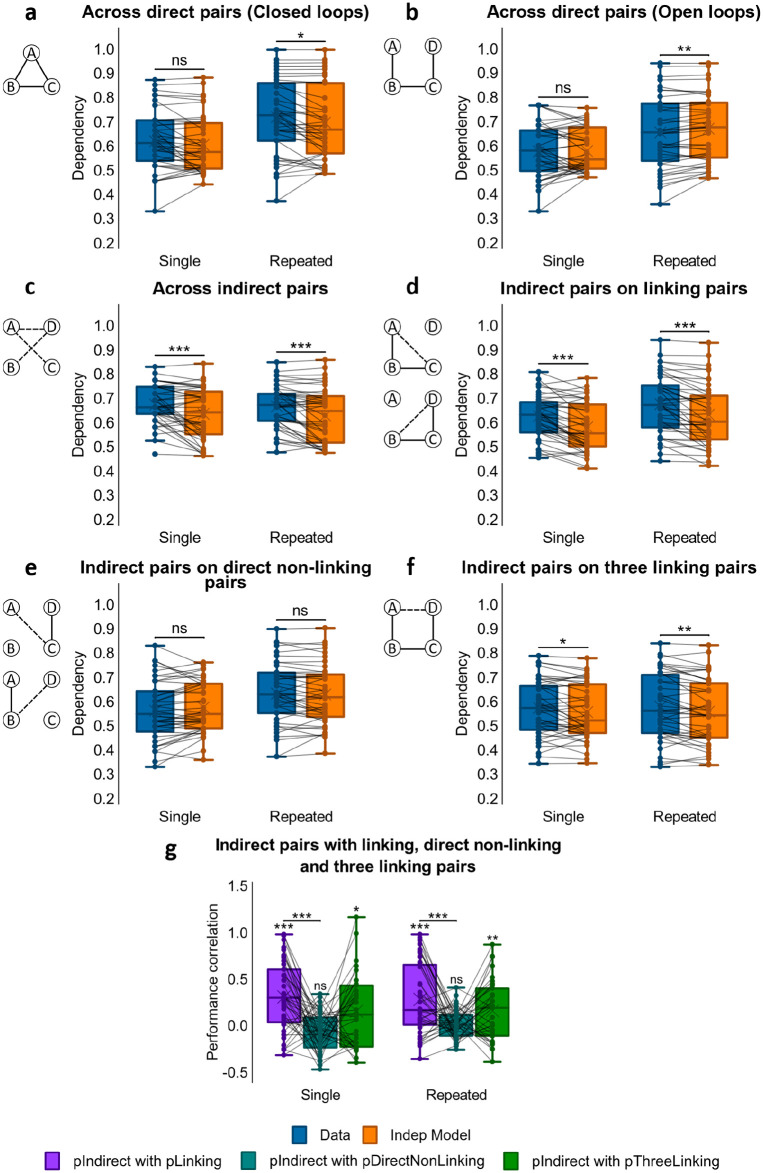
Dependency results for Experiment 3. (a) Dependency of direct pairs on other direct pairs from the same event for Single Closed and Repeated Closed loops, and corresponding Independent model. (**b**) Dependency of direct pairs on other direct pairs from the same event for Single Open and Repeated Open loops, and corresponding Independent model. (**c**) Dependency of indirect pairs on other indirect pairs from the same event for Single Open and Repeated Open loops, and corresponding Independent model. (**d**) Dependency of indirect pairs on related direct pairs from the same event for Single Open and Repeated Open loops, and corresponding Independent model. (**e**) Dependency of indirect pair on unrelated direct pairs from the same event for Single Open and Repeated Open loops, and corresponding Independent model. (**f**) Dependency of indirect pair AD on all direct linking pairs from the same event for Single Open and Repeated Open, and corresponding Independent model. (**g**) Performance correlation between indirect associations and direct linking, direct non-linking (for AC and BD) and three linking direct associations (for AD), in single and repeated events. ^***^p < 0.001; ^**^p < 0.01; *p < .05; ns not significant. *N* = 42 for **a**-**g**.

As in Experiments 1 and 2, we next examined dependency across loop-type and repetition (see Figure 7a and b). A 2x2 repeated measures ANOVA (loop-type x repetition) showed a main effect of loop-type, *F*(1, 41) = 7.12, *p* = .011, η_*p*_^2^ = .148, but no significant effects of repetition, *F*(1, 41) = .035, *p* = .853, or loop-type x repetition interactions, *F*(1, 41) = 1.43, *p* = .238.

To further assess the effect of loop-type, we separately analysed closed- and open-loop structures, looking for differences in dependency using a post hoc paired samples *t*-test. Consistent with results from Experiments 1 and 2, closed-loop events showed greater dependency than open-loop events, *t*(41) = 2.68, *p* = .011, *d* = .414. Subsequent one-sample *t*-tests reported dependency in closed-loop structures, *t*(41) = 4.25, *p* < .001, *d* = .655, but not in open-loop ones, *t*(41) = 0.306, *p* = .761.

#### Dependency across indirect associations

We next looked at dependency across all three indirect associations (i.e., AC, BD, and AD) for open-loop events (see Figure 7c). Analysis using a one-way ANOVA showed no main effect of repetition, *F*(1, 41) = 0.006, *p* = .940, hence no difference in dependency between single and repeated conditions.

The amount of within-event dependency across inferred associations was then compared with the amount of within-event dependency across direct associations. A 2x2 ANOVA (direct vs. indirect x repetition) reported a main effect of association-type, *F*(1, 41) = 20.3, *p* < .001, η_*p*_^2^ = .331, but no effect of repetition, *F*(1, 41) = 0.926, *p* = .341, and no interaction, *F*(1, 41) = 0.389, *p* = .536. To further study the difference in dependency, we ran a paired samples *t*-test comparing dependency across direct associations with dependency across indirect analyses and observed greater dependency among indirect associations than among direct associations in open-loop events, *t*(41) = −3.11, *p* = .003 *d* = −.480.

#### Dependency of indirect associations on linking direct associations

We next examined whether the retrieval of an indirect association was dependent on the successful retrieval of the two linking direct associations that would be required to make an inference (i.e., whether retrieval of AC was more likely given the successful retrieval of AB and BC, and of BD given successful retrieval of BC and CD; see Figure 1b for an illustration of the event structure; see Figure 7d). A one-way ANOVA showed no main effect of repetition, *F*(1, 41) = 0.030, *p* = .863, and studying the findings further using a one-sample *t*-test, we saw significant dependency across both single and repeated events, *t*(41) = 6.68, *p* < .001, *d* = 1.03. This suggested that the ability to retrieve indirect associations relied on the success of direct associations that would be required to make an inference.

The relatedness in performance between indirect associations and linking associations across events was further examined by computing Pearson correlation coefficients between participants’ accuracy scores for the indirect associations and the product of their accuracy scores for the linking pairs across events (see Figure 7g). Fisher’s *Z*-transformed *r* values were significantly greater than zero for both single open-loop events, mean *r* = .265, *t*(41) = 5.64, *p* < .001, *d* = .871, and repeated open-loop events, mean *r* = .253, *t*(40) = 4.85, *p* < .001, *d* = .757. In a paired samples *t*-test comparing the average transformed correlation coefficients in single and in repeated events, we saw no difference, *t*(40) = 0.384, *p* = .703. Thus, the retrieval of inferred associations was related across open-loop events to the retrieval of linking associations, and this correlation was not affected by repetition.

#### Dependency of indirect associations on direct non-linking associations

Next we asked whether retrieval of the indirect associations AC and BD was dependent on the retrieval of direct non-linking associations that would not be expected to support inference (see Figure 1b for an illustration of the event structure; see Figure 7e). Specifically, we looked at the dependency between retrieving the indirect association AC and the direct association CD, and the dependency between retrieving the indirect association BD and the direct association AB. A one-way ANOVA showed an effect of repetition that almost reached significance, *F*(1, 41) = 3.99, *p* = .052, η_*p*_^2^ = .089. However, subsequent one-sample *t*-tests showed no dependency in either the single, *t*(41) = −1.98, *p* = .055, or repeated presentation condition, *t*(41) = .525, *p* = .603, although the former was approaching significance. Thus, there was no evidence for dependency between indirect associations and direct non-linking associations.

We then compared dependency for indirect associations on direct linking associations and for indirect associations on direct non-linking associations. A 2x2 (repetition x linking vs. non-linking) ANOVA demonstrated a significant main effect of dependency analysis, *F*(1, 41) = 27.2, *p* < .001, η_*p*_^2^ = .399, but no main effect of repetition, *F*(1, 41) = 2.19, *p* = .147, nor interaction between repetition and dependency analysis, *F*(1, 41) = 2.14, *p* = .151. A paired samples *t*-test comparing the amount of dependency for indirect associations on linking direct pairs and on direct non-linking pairs showed a significant difference, *t*(41) = 4.88, *p* < .001, *d* = .753, where inferred associations in both repetition conditions had stronger dependency on linking pairs than on direct non-linking pairs.

To probe the relationship between the retrieval of inferred and non-linking associations across events, each participant’s performance on indirect associations was correlated with the product of performance on the direct non-linking associations (see Figure 7g). Fisher’s Z-transformed *r* values for both single, mean *r* = −.056; *t*(41) = −1.74, *p* = .090, and repeated events, mean *r* = .024; *t*(41) = 1.10, *p* = .278, did not differ from zero. In addition, a paired samples *t*-test comparing transformed *r* values between single and repeated open-loop events revealed a significant difference favouring the latter, *t*(41) = −2.03, *p* = .049, *d* = −.313. Hence, although repetition had a positive effect on the correlation, accuracy for indirect associations was not related to performance for non-linking pairs across both single and repeated open-structure events.

#### Dependency of indirect association AD on all linking direct associations

We later examined whether the retrieval of the indirect association AD was dependent on the retrieval success of all linking direct associations that would be required to form an associative chain to aid its inference (i.e., AB-BC-CD, see Figure 1b for an illustration of the event structure; see Figure 7f). Using a one-way ANOVA, we saw no main effect of repetition, *F*(1, 41) = .209, *p* = .650, but a one-sample *t*-test showed dependency of AD on all direct linking pairs, *t*(41) = 3.87, *p* < .001, *d* = .596.

The relationship between the retrieval of AD and of all linking direct associations across open-loop events was then further examined by correlating participants’ accuracy scores for AD and the product of accuracy scores for AB, BC, and CD across events (see Figure 7g). Fisher’s Z-transformed *r* values were significantly greater than zero for both single, mean *r* = .135; *t*(33) = 2.41, *p* = .021, *d* = .414, and repeated events, mean *r* = .151; *t*(32) = 3.01, *p* = .005, *d* = .524, with no significant difference between the two, *t*(30) = .037, *p* = .971. These results suggested that the successful retrieval of AD was linked to the successful retrieval of the entire associative chain of AB-BC-CD across events, unaffected by encoding repetition.

### Summary of Experiment 3

Although Experiment 3 introduced encoding repetition, which improved performance, the dependency results remained consistent with those from Experiments 1 and 2. Significant dependency among direct associations was seen within closed-loop events but not within open-loop events. Nonetheless, as in Experiments 1 and 2, inferred associations within open-loop events were dependent on each other. The retrieval of indirect associations also displayed dependency on that of direct linking, but not non-linking, associations; and performance on inference trials correlated with the product of performance on the direct linking associations. Like Experiment 2, dependency across inferred associations was greater than across direct associations, while there was no such difference in Experiment 1. The awareness of the type of association to be tested in an upcoming trial in Experiments 2 and 3, as well as the testing of direct associations before indirect ones, perhaps contributed to the more robust dependency between inferred than between direct associations. As in Experiment 2, retrieving solely direct associations prior to indirect associations could have enhanced memory of the former such that inferences made across them were retrieved in an interdependent manner. Across events, memory of indirect associations was related to memory of linking direct associations, but not with direct non-linking associations.

Importantly, repeating the presentation of events yielded no change in either within-event dependency or performance correlation across events. Hence, although encoding repetition significantly strengthened memory of learned information, it did not help integrate encoded associations into a coherent representation of the event.

Our results once again endorse the idea that both directly learned and inferred pairs were encoded and retrieved from a unitary associative network, enabling events to be comprehensively retrieved. While direct associations in open-loop events did not involve pattern completion, inference across those associations did show evidence of pattern completion as a retrieval mechanism via a dependence on the retrieval of encoded linking associations.

## Experiment 4—a computational model of hippocampal pattern completion during inference

All three experiments described above produced similar results—showing significant dependency among direct associations in closed-loop but not open-loop events, among inferred associations in open-loop events, and among inferred associations and their direct linking pairs in open-loop events. To establish whether these findings could be accounted for by a canonical computational account of hippocampal memory function, we next simulated a simple auto-associative neural network model (adapted from Horner et al., 2015). First, the network probabilistically encoded a series of overlapping pairwise associations presented either one or three times, equivalent to the empirical protocol in Experiment 3. During subsequent retrieval, a single “cue” neuron was externally stimulated while six other “target” neurons, corresponding to the forced choice alternatives, were partially activated. Firing rates in each of the target neurons, which could be boosted by recurrent connectivity, were then inspected to establish whether successful retrieval had occurred (as indicated by firing rates exceeding a specified threshold), and accuracy and dependency for each event and pair-type were analysed as described above. This allows us to establish whether the behavioural findings described by Experiments 1–3 could each be accounted for by a process of hippocampal pattern completion.

### Method

We simulated a network of N rate-coded neurons (Equation 2) that were fully recurrently connected except for self-connections (adapted from Horner et al., 2015). The firing rate $r_{i}$ of these neurons was dictated by a time constant $\tau_{r}$ = 25 ms, a combination of externally applied currents $I_{i,ext}$ and recurrent synaptic currents $I_{i,syn}$ , and a sigmoidal transfer function (Equation 3). We parameterized the transfer function with a threshold $r_{t}$ = 10 and a peak firing rate of $r_{max}$ = 10 Hz. All firing rates $r_{i}$ and synaptic connections $w_{ij}$ within the network were initially set to zero.

(2) $$\tau_{r}\frac{dr_{i}}{dt}=-r_{i}+f\left(I_{i,ext}+I_{i,syn}\right)$$

(3) $$f\left(x\right)=\frac{r_{max}}{1+\exp\left(r_{t}-x\right)}$$

Each element of an event was represented by a unique neuron, and the encoding order and resulting associative structures for the closed-loop and open-loop conditions were identical to Experiment 3. During encoding, we assumed that synaptic connections with a strength of $w_{ij}$ = 1.1 were formed between neurons representing the pair of stimuli being presented in each trial with a probability of $p_{enc}$ . To account for variance in performance across simulated participants, values of $p_{enc}$ for each simulation were chosen randomly from a Gaussian distribution with a mean of $\mu_{enc}$ = 0.3 and a standard deviation of $\sigma_{enc}$ = 0.2. Where pairs of stimuli were repeated, synaptic connections were only updated if they had a strength of $w_{ij}$ = 0 (i.e., there was no “forgetting,” where synaptic connections formed in earlier encoding blocks were eliminated), such that the overall proportion of potentiated synaptic connections increased across blocks.

The retrieval order for each pairwise association in the closed-loop and open-loop conditions was identical to the main experiment. During retrieval, the neuron that represented the cued element received a constant current $I_{ext}$ = 15 for a period of $t_{ret}$ = 1 s, while neurons that represented the six forced choice target elements received a constant current of $I_{ext}$ = 6. Additional activity was generated by the recurrent synaptic current $I_{syn}$ , which is the product of the synaptic weights and firing rates of connected neurons (Equation 4).

(4) $$I_{i,syn}=\sum_{j}w_{ij}r_{j}$$

To convert firing rates in a retrieval trial into performance on that trial, we looked for neurons representing the six forced choice target elements whose firing rate at the end of the trial exceeded a retrieval threshold of $r_{ret}$ = 8 Hz. If the activity of multiple neurons exceeded this threshold, then we selected one at random to determine the simulated response. Conversely, if no neurons exceeded the retrieval threshold at the end of the trial, then the simulated response was chosen at random from the six forced choice target elements.

Finally, associative accuracy and statistical dependency were computed as described above in relation to the behavioural data. A total of 43 simulations were performed (to match the number of participants in Experiment 3), each containing 60 events (30 closed-loop and 30 open-loop, with 18 events from each condition being repeated three times during encoding).

After conducting a Shapiro-Wilk test of normality on Ddata-Di analyses, a deviation from a normal distribution was found in dependency across direct pairs in repeated closed events, *W*(43) = .869, *p* < .001, single open events, *W*(43) = .945, *p* = .038, repeated open events, *W*(43) = .944, *p* = .035, and repeated events, *W*(43) = .909, *p* = .002, as well as dependency across indirect pairs in single open events, *W*(43) = .911, *p* = .003, and repeated open events, *W*(43) = .943, *p* = .032, dependency of indirect pairs on direct linking pairs in single open events, *W*(43) = .939, *p* = .024, dependency of indirect pairs on direct non-linking pairs in single open events, *W*(43) = .939, *p* = .024, and dependency of AD on all linking pairs in single open events, *W*(43) = .910, *p* = .002. All dependency analyses thus underwent a log transformation (Equation 1) before statistical tests were performed.

### Results

#### Associative accuracy

**Figure 8.**
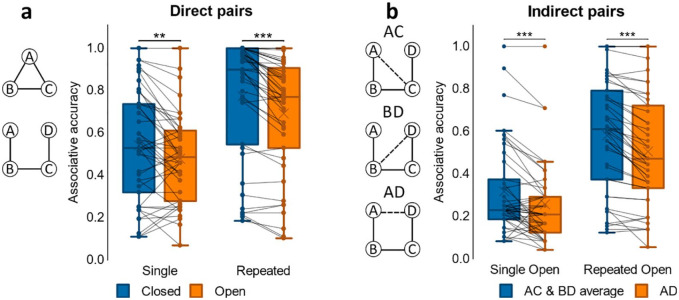
Simulated associative accuracy results (Experiment 4). (a) Proportion correct retrievals in Single and Repeated events for direct pairs in closed and open loops. (**b**) Proportion correct retrievals in Single and Repeated events for indirect pairs AC, BD, and AD. ***p < .001; **p < .01. *N* = 43 for both a and b.

To examine associative accuracy (see Figure 8a) for direct associations (i.e., those shown during encoding), performance for single and repeated events in closed- and open-loop structures was computed. A 2x2 ANOVA (loop-type, repetition) revealed a main effect of loop-type, *F*(1, 42) = 30.4, *p* < .001, η_*p*_^2^ = .420, resulting from better memory performance for closed- compared to open-loop events. This analysis also revealed a main effect of repetition, *F*(1, 42) = 121.1, *p* < .001, η_*p*_^2^ = .742, caused by better performance for repeated pairs than for those presented only once during the study phase. There was no significant interaction between loop-type and repetition, *F*(1, 42) = .925, *p* = .342.

Next, we compared associative accuracy between direct and indirect associations in open-loop events. A 2x2 ANOVA (association-type x repetition) revealed a significant interaction, *F*(1, 42) = 7.69, *p* = .008, η_*p*_^2^ = .155, as well as main effects of both association-type, *F*(1, 42) = 151.5, *p* < .001, η_*p*_^2^ = .783, and repetition, *F*(1, 42) = 102.0, *p* < .001, η_*p*_^2^ = .708, due to better memory performance for direct associations and for repeated events. Post hoc paired-sample *t*-tests revealed that accuracy of direct associations in open-loop events was greater with encoding repetition, *t*(42) = 9.83, *p* < .001, *d* = 1.50, and the same was seen for indirect associations, *t*(42) = 9.42, *p* < .001, *d* = 1.44. In single open-loop events, performance in direct pairs was greater than in indirect pairs, *t*(42) = 11.1, *p* < .001, *d* = 1.69, likewise in repeated open-loop events, *t*(42) = 9.62, *p* < .001, *d* = 1.47. The interaction was driven by greater difference in accuracy between direct and inferred associations in repeated presentation than in single events, *t*(42) = 2.77, *p* = .008, *d* = .423.

Next, we examined performance across indirect associations from open-loop structures (i.e., AC, BD, AD; see Figure 8b). A 3x2 ANOVA (pair-type, repetition) demonstrated a main effect of pair-type, *F*(2, 84) = 21.4, *p* < .001, η_*p*_^2^ = .337, as a result of better associative accuracy for AC, *t*(42) = 5.95, *p* < .001, *d* = .908, and BD associations, *t*(42) = 5.62, *p* < .001, *d* = .858, versus AD. Accuracy for AC and BD did not vary, *t*(42) = .891, *p* = .378. In addition, we observed a main effect of repetition, *F*(1, 42) = 88.5, *p* < .001, η_*p*_^2^ = .678, due to higher performance for repeated associations, but no significant interaction, *F*(2, 84) = .802, *p* = .452. Overall, repetition had a beneficial effect on accuracy in both direct and indirect associations, and performance for indirect pairs AC and BD were not just similar to each other, but separately better than for the indirect pair AD.

#### Dependency across direct associations

**Figure 9.**
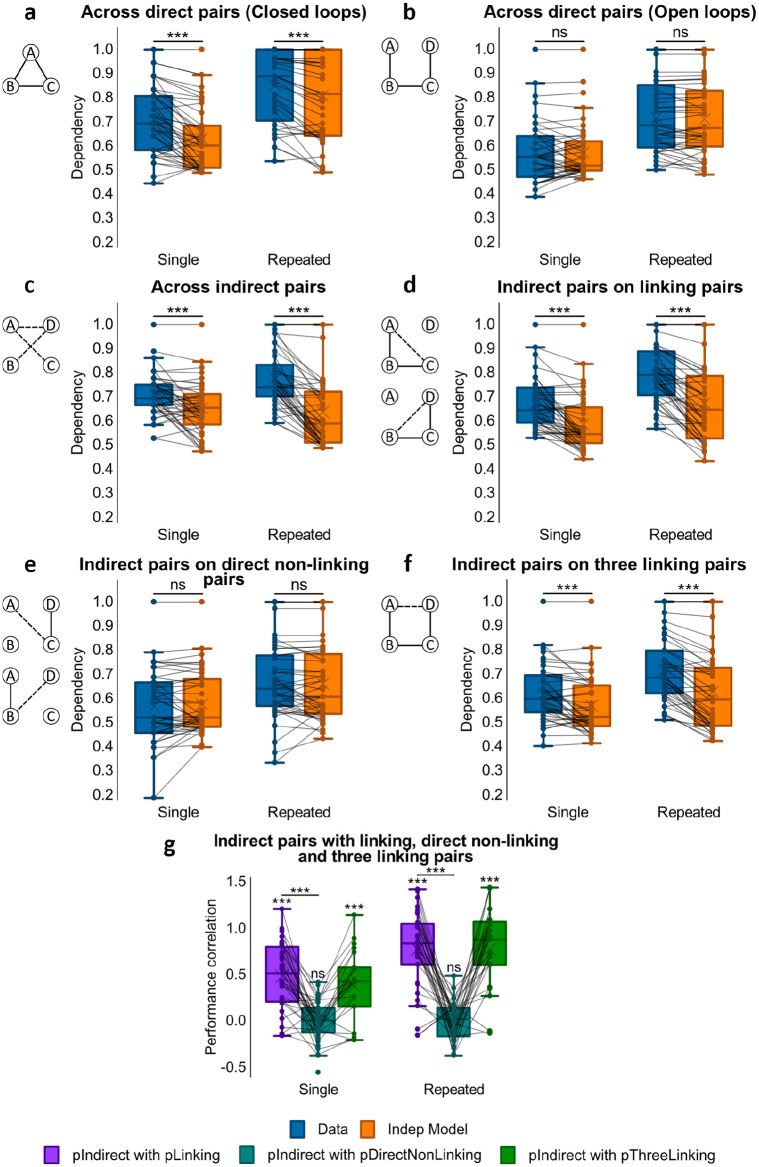
Simulated dependency results (Experiment 4). (a) Dependency of direct pairs on other direct pairs from the same event for Single Closed and Repeated Closed loops, and corresponding Independent model. (**b**) Dependency of direct pairs on other direct pairs from the same event for Single Open and Repeated Closed loops, and corresponding Independent model. (**c**) Dependency of indirect pairs on other indirect pairs from the same event for Single Open and Repeated Closed loops, and corresponding Independent model. (**d**) Dependency of indirect pairs on related direct pairs from the same event for Single Open and Repeated Closed loops, and corresponding Independent model. (**e**) Dependency of indirect pair on unrelated direct pairs from the same event for Single Open and Repeated Closed loops, and corresponding Independent model. (**f**) Dependency of indirect pair AD on all direct linking pairs from the same event for Single Open and Repeated Closed, and corresponding Independent model. (**g**) Performance correlation between indirect associations and linking direct, direct non-linking (for AC and BD) and three linking direct associations (for AD), in single and repeated events. ^***^p < .001; ns: not significant. *N* = 43 for **a**-**g**.

Next, we examined dependency in the data for direct associations in contrast to estimates from the Independent model across loop-type and repetition (see Figure 9a and b), as for the empirical data from Experiments 1–3. We performed a 2x2 repeated measures ANOVA (loop-type, repetition) and found evidence of a loop-type x repetition interaction, *F*(1, 42) = 4.85, *p* = .033, η_*p*_^2^ = .104, and a main effect of loop-type, *F*(1, 42) = 41.5, *p* < .001, η_*p*_^2^ = .497, but not repetition, *F*(1, 42) = 2.47, *p* = .123.

To probe the loop-type x repetition interaction, we used paired samples *t*-tests to look at dependency in closed- and open-loop structures. Closed-loop events showed stronger dependency than open-loop ones, *t*(42) = 6.45, *p* < .001, *d* = .984, this difference being significantly larger in single loops than in repeated loops, *t*(42) = 2.20, *p* = .033, *d* = .336. Following further one-sample *t*-tests to assess dependency, we observed dependency in closed-loop events, *t*(42) = 8.00, *p* < .001, d = 1.22, which was absent in open-loop events, *t*(42) = .718, *p* = .477. These results were similar to findings from Experiments 1, 2, and 3.

#### Dependency across indirect associations

To assess the dependency of indirect associations (i.e., AC, BD, and AD) from the same open-loop event on each other (see Figure 9c), we used a one-way ANOVA. Analyses revealed a main effect of repetition, *F*(1, 42) = 19.0, *p* < .001, η_*p*_^2^ = .312, and post hoc paired samples *t*-tests reported dependency significantly increasing with repetition, *t*(42) = −4.36, *p* < .001, *d* = −.665. A one-sample *t*-test showed dependency across inferred associations within open-loop events, *t*(42) = 8.37, *p* < .001, *d* = 1.28.

Next, we compared the amount of dependency across inferred associations to that across direct associations within open-loop events. Using a 2x2 ANOVA (direct vs. indirect analysis x repetition), we saw a main effect of type of dependency analysis, *F*(1, 42) = 63.8, *p* < .001, η_*p*_^2^ = .603, and repetition, *F*(1, 42) = 14.1, *p* = .001, η_*p*_^2^ = .252, as well as a significant interaction between the two, *F*(1, 42) = 16.1, *p* < .001, η_*p*_^2^ = .278. Post hoc *t*-tests indicated that these effects were driven by the dependency across indirect associations being significantly greater than across direct associations for both single, *t*(42) = 3.81, *p* < .001, *d* = .581, and repeated events, *t*(42) = 8.39, *p* < .001, *d* = 1.28, with the magnitude of the difference being greater in the latter case. Overall, we noted higher dependency among indirect associations than among direct associations within the same open-loop event.

#### Dependency of indirect associations on direct linking associations

We then measured the dependency of indirect associations on the accurate retrieval of the two linking direct associations necessary to make the inference (i.e., between the retrieval of AC and that of AB and BC, and the retrieval of BD and that of BC and CD; see Figure 1b for an illustration of the event structure; see Figure 9d). Analysis using a one-way ANOVA showed no main effect of repetition, *F*(1, 42) = 2.76, *p* = .104. A one-sample *t*-test indicated dependency, *t*(42) = 8.37, *p* < .001, *d* = 1.28, implying that retrieving indirect associations relied on the successful retrieval of direct associations that would be required to make an inference.

To further probe the relationship between performance on indirect associations and their linking associations across events, we computed Pearson correlation coefficients between accuracy for the indirect associations and the product of accuracy scores for the linking pairs across events (see Figure 9g). The resulting Fisher’s Z-transformed *r* values were greater than zero both for single open-loop events, mean *r* = .431; *t*(34) = 8.15, *p* < .001, *d* = 1.38, and repeated open-loop events, mean *r* =.626; *t*(34) = 11.0, *p* < .001, *d* = 1.86, with the latter significantly greater than the former, *t*(29) = −5.17, *p* < .001, *d* = −.944. Hence, the retrieval of inferred associations was correlated with the retrieval of linking associations across open-loop events, this correlation enhanced by encoding repetition.

#### Dependency of indirect associations on direct non-linking associations

Retrieval of the indirect associations AC and BD was then analysed for evidence of dependency on the retrieval of direct non-linking associations, which were not expected to be pertinent when making an inference (see Figure 1b for an illustration of the event structure; see Figure 9e). Specifically, we measured the dependency of retrieving the indirect pair AC on retrieval of the direct pair CD, and the dependency of retrieving the indirect pair BD on retrieval of the direct pair AB. A one-way ANOVA demonstrated no effect of repetition, *F*(1, 42) = 1.90, *p* = .175, and a one-sample *t*-test indicated no dependency, *t*(42) = .119, *p* = .906. Therefore, no dependency between indirect associations and direct non-linking associations was observed.

Next we compared the amount of dependency of indirect associations on direct linking and non-linking associations. Results from a 2x2 (repetition x direct linking vs. direct non-linking analysis) ANOVA revealed a significant main effect of type of dependency analysis, *F*(1, 42) = 88.5, *p* < .001, η_*p*_^2^ = .678, and a main effect of repetition that tended towards significance, *F*(1, 42) = 3.97, *p* = .053, η_*p*_^2^ = .086, but no interaction, *F*(1, 42) = .254, *p* = .617. Post hoc paired samples *t*-tests revealed that indirect associations had significantly greater dependency on direct linking pairs and that on direct non-linking pairs, *t*(42) = 9.41, *p* < .001, *d* = 1.44, suggesting that inferred associations exhibited greater dependency on linking pairs than on direct non-linking pairs.

To further probe the relationship between the retrieval of indirect associations and non-linking associations across events, we correlated performance for indirect associations with the product of performances for the direct non-linking associations (see Figure 9g). As seen in Experiment 3, Fisher’s Z-transformed *r* values were not different from zero for either single, mean *r* = .001; *t*(41) = −.0003, *p* > .99, or repeated events, mean *r* = .005; *t*(37) = .161, *p* = .873. We also noted no significant difference in Z-transformed *r* values between single and repeated open-loop events, *t*(37) = −.133, *p* = .895. These results suggest that there is no relationship between accuracy for indirect associations and accuracy for direct non-linking pairs across events, with repetition having no influence on this correlation.

#### Dependency of indirect association AD on all linking direct associations

We subsequently assessed how dependent the indirect association AD was on retrieval of all the direct linking associations in the associative chain that might support its inference (i.e., AB-BC-CD, see Figure 1b for an illustration of the event structure; see Figure 9f). A one-way ANOVA found a main effect of repetition, *F*(1, 42) = 7.83, *p* = .008, η_*p*_^2^ = .157, and upon further examination using a paired samples *t*-test, we saw greater dependency in repeated than in single presentations, *t*(42) = −2.80, *p* = .008, *d* = −.427. A one-sample *t*-test found dependency in all open events, *t*(42) = 9.47, *p* < .001, *d* = 1.44. Hence, inferred association AD was dependent for retrieval on the retrieval of all direct linking pairs, more so in repeated events.

To further probe the relationship between the retrieval of AD and that of all direct linking associations, we correlated accuracy scores for AD with the product of accuracy scores for AB, BC and CD across open-loop events (see Figure 9g). As in Experiment 3, we found that Fisher’s Z-transformed *r* values were greater than zero for both single, mean *r* = .430; *t*(21) = 4.66, *p* < .001, *d* = .994, and repeated events, mean *r* = .674; *t*(26) = 9.11, *p* < .001, *d* = 1.75. However, unlike Experiment 3, there was a significant change in Z-transformed *r* values with encoding repetition, *t*(13) = −2.68, *p* = .019, *d* = −.717. Successfully retrieving AD was hence linked to successfully retrieving the associative chain of AB-BC-CD across events, and repetition had a beneficial effect on this correlation.

### Summary of simulations (Experiment 4)

The results described above indicate that a simple computational model of hippocampal memory function produces a similar pattern of retrieval accuracy and dependency as the empirical findings from Experiments 1, 2, and 3. As with our experiments, retrievals of overlapping associations from closed- but not open-loop events were dependent on each other. We also saw significant retrieval dependency among indirect associations from the same open-loop event. The successful retrieval of these indirect associations appeared to rely on that of direct linking, but not non-linking, associations. Similarly, performance on indirect associations across events correlated with the product of performance on direct linking pairs, as observed in Experiments 1, 2 and 3, with repetition strengthening such correlations. Inferred associations had greater within-event dependency than direct associations (as seen in Experiments 2 and 3 but not Experiment 1). Finally, encoding repetition improved performance on direct (observed) and indirect associations (as in Experiment 3), and also increased the dependency of indirect associations (which was not seen in Experiment 3).

Our simulations demonstrate that the main pattern of experimental results can be accounted for by a process of pattern completion in an auto-associative neural network (with one exception, discussed below), and are therefore broadly consistent with contemporary models of hippocampal memory function.

## General discussion

Here, we examined the associative structure of encoded events and their contribution to successful inference across unseen associations. Across three experiments, we found no evidence for statistical dependency in the retrievals of pairwise associations from the same partially observed “open-loop” events, in contrast to the dependency found for retrievals from the same fully observed “closed-loop” events. These findings replicate previous experiments (Horner & Burgess, 2013, 2014; Horner et al., 2015) and suggest that a process of holistic pattern completion occurs for closed loops of overlapping pairs, but not for open loops. In addition, it extends previous results by showing that repeated presentation of the associations in open loops improves memory for the associations but does not increase dependency between them.

However, inferences for unseen associations in open-loop events (i.e., AC, BD, or AD in the chain AB, BC, and CD) were highly dependent on retrieval of other observed or unseen associations from the same event. This interdependency likely reflects a mechanism of pattern completion that is used for inferring indirect associations from partially observed open-loop events and also for retrievals from fully observed closed-loop events. We speculate that retrievals of observed associations may reflect either recollection of the individual presentation of that association (independent of other overlapping associations), or pattern completion in which all overlapping associations are retrieved. In this view, open-loop events afford individual retrievals while closed-loop events afford pattern completion due to the greater number of associations through which activity can spread (e.g., from A to B via AB and also via AC-BC). When inferring unseen associations, recollection of the presentation of the association is not possible, so pattern completion must be used. This will involve activity spreading via the observed linking associations (e.g., inferring AC via AB and BC), explaining the correlation between inference performance and the product of performances on the linking associations. The interdependency among unseen associations within open loops appears to reflect the fact that they share a common observed linking association (i.e., BC). Thus, the retrievals of different inferential associations from an open loop event all depend on the successful retrieval of the same direct linking association.

In the empirical data, repetition had no strong effect on dependency for either closed or open loops, direct or indirect associations, suggesting that simply strengthening the open-loop associations in this way is not sufficient to induce pattern completion and thus holistic representation of the open loops of direct associations. Thus, increased associative strengths (and increased numbers of individual presentations that could be recollected) did not strongly affect the likelihood of pattern completion relative to individual recollection of presentation when retrieving a specific paired associate (whereas inferring an indirect association can only occur via pattern completion).

Consistent with our interpretation, a computational model of the hippocampus as an auto-associative network replicated our main pattern of findings regarding accuracy and dependency, indicating that even though “open loops” of overlapping pairwise associations were retrieved independently, inferences made across them were reliant on the linking associations that enable the inference.

The model differed from the data in showing an increase in dependency among indirect associations when presentation of the direct associations forming open loops were repeated, concomitant with an increase in accuracy. This arises from a reduction in the overall proportion of answers that are guesses (and will be independent) relative to the proportion that can be accounted for by pattern completion (which will show dependency), which increases overall dependency. It is not clear why this effect was not seen in the empirical data, and this is a topic for future experiments.

Previous research has indicated that both encoding and retrieval processes potentially underlie inference. While some have suggested that events are stored as independent memory traces and then recalled and recombined at retrieval to support transfer (Banino et al., 2016; Kumaran & McClelland, 2012; Wu & Levy, 2001), others propose dynamic learning interactions during which overlapping past events are stored as integrated mnemonic representations (Howard et al., 2005; O’Reilly & Rudy, 2000; Shohamy & Wagner, 2008). Our model assumes that inference results from pattern completion via the relevant direct linking associations. However, our current results cannot specify whether this occurs purely during retrieval, or whether there is some pattern completion and learning of indirect associations during or shortly after the encoding of the direct associations. All we can say is that, if indirect associations are partially formed prior to the retrieval tests, they are too weak to support dependency between direct associations from open-loop events, and are thus weaker than the direct associations formed in closed-loop events. To more accurately identify the point at which inferences are forged will require further experimental manipulations.

The retrieval dependency of indirect associations highlights the reconstructive nature of episodic memory, comprising not just the storage of information but the flexible inference of acquired knowledge. For open-loop events, the inference of unseen associations appears to have been achieved by pattern completion via observed linking associations, even though there was no evidence for pattern completion during their own retrieval. This is consistent with a retrieve-and-integrate interpretation of associative inference (Banino et al., 2016; Carpenter & Schacter, 2017; Kumaran & McClelland, 2012; Schacter & Addis, 2007a, 2007b; Wu & Levy, 2001; Zeithamova et al., 2012), in which independent associations can be retrieved and used to support pattern completion to solve the inference task.

The rapid formation of new long-term memories is usually thought to depend on the hippocampus, which then enables slow formation of semantic knowledge in neocortical areas (Marr, 1971; McClelland et al., 1995; Scoville & Milner, 1957; Tulving, 1985; but see also Squire & Zola-Morgan, 1991). However, where new knowledge is consistent with, and incremental to, previously learned knowledge (or “schema”), it can be integrated directly into the neocortical system (Tse et al., 2007). Computational modelling suggests that this integration only requires limited reactivation of related data (McClelland et al., 2020). Thus, our evidence relating associative inference (AC, BD) to pattern completion via existing associations (AB, BC, CD) might reflect neocortical integration as well as hippocampal associative memory.

A point of discussion is whether overlapping pairwise associations can be considered as separate episodic events or as associations within the same extended episode. In previous work (Horner & Burgess, 2013, 2014; Horner et al., 2015), closed loops of overlapping pairwise associates were considered to belong to the same episode despite being encoded at different times, showing the same dependency across retrievals as for simultaneously encoded events (Horner & Burgess, 2013). However, open loops did not show this dependency, here or in previous work (Horner & Burgess, 2013, 2014; Horner et al., 2015). On these grounds, they should not be considered as forming “events,” following previous work on pairs of overlapping associations (Banino et al., 2016; Schlichting et al., 2014; Shohamy & Adcock, 2010; Shohamy & Wagner, 2008; Zeithamova et al., 2012, 2016). In this view, it is the presence of pattern completion and resulting statistical dependency that determines whether separate occurrences become distinct episodic memories. Our results, showing that inferred associations from open loops of overlapping pairs did show statistical dependency on each other and on the direct linking pairs, raise questions for this dichotomy. They imply that pattern completion can be triggered, either by a set of associative connections (closed loops) or by requiring associative inference which in turn can be solved by pattern completion more readily via the linking direct associations or via other inferential judgements from the same event.

Inferential reasoning in our experiments had been licenced by the experimenter as participants were specifically told to look out for any indirect links between the cue and the test options. In real life, however, congruent episodic events might not necessarily give rise to such inferences. In reality, seeing Barack Obama in the kitchen one moment and later a hammer in the kitchen might not always lead one to relate Obama to the hammer. Numerous factors ranging from memory interference (Anderson & Neely, 1996; Robertson, 2012; Shapiro & Olton, 1994) to context (Bransford & Johnson, 1972; Godden & Baddeley, 1980; Smith & Vela, 2001), schemas (Ghosh & Gilboa, 2014; Tse et al., 2007), and prior knowledge (Alba & Hasher, 1983; Preston & Eichenbaum, 2013; van Kesteren et al., 2010; Wang & Morris, 2010) will also affect the inferential process. One potential future study could explore inference construction when it is explicitly licenced, when there is no mention of it in the instructions, and when participants are advised against making unfounded inferential presumptions across complementary associations.

Reaction time (RT) analysis could potentially yield interesting insights regarding the processes supporting retrieval of direct associations in closed versus open loops and retrieval of indirect and direct associations within open loops. Accordingly, we analysed RTs in Experiment 1 (in which retrievals of direct and indirect associations were interleaved within the same session). We did not find differences in RT for retrieving direct associations from closed versus open loop events, *F*(1, 24) = 0 .218, *p* = .645. We did find slower RTs for retrieving indirect versus direct associations from open loop events, *F*(1, 24) = 42.1, *p* < .001, η_*p*_^2^ = .637. However, the interpretation of this result is not straightforward, as performance is worse for indirect than direct associations. Similarly, Experiment 3 (which also showed no differences in RTs when retrieving direct associations from closed versus open loops) showed faster RTs for retrieval of repeated versus singly presented associations, *F*(1, 42) = 40.8, *p* < .001, η_*p*_^2^ = .493. Both results might potentially reflect performance levels rather than process differences.

In Experiment 3, where repetition was assessed, single trials on average had a greater interval between their final encoding trial and test compared to repeated trials, which might decrease memory performance for single events. The last trial of each repeated event was presented in the last block (see Experiment 3—Methods) while that was not necessarily the case for the last trial of each single-presented event. Future studies could attempt to equate this interval in both conditions by presenting encoding trials for single events during the last block.

Our results overall provide evidence that inferred information makes use of hippocampal pattern completion for retrieval even if the process was not engaged during the retrieval of encoded associations. Interleaving retrieval of directly encoded and inferred associations and testing the latter before the former produced the same observations on dependency as separating them and testing direct associations first. However, the latter manipulations can boost dependency and thus pattern completion among inferred associations. Repetition had no impact on pattern completion among direct associations in open loops (A-B-C-D) despite increasing the likelihood of correctly retrieving inferred AD pairs that “closed” the loops, suggesting that retrieval of inferred knowledge is different from the encoding of observed associations in terms of its effect on pattern completion. Whether this difference is qualitative or quantitative (inferred associations being weaker) remains a topic for the future.

In conclusion, we show that although overlapping associations encoded in an open loop can be retrieved independently, unseen associations inferred across them are significantly dependent on the retrieval of relevant encoded associations from the same event. Moreover, this dependency on directly encoded associations produces dependency between inferred indirect associations from the same event. The findings suggest that both directly learned and indirectly inferred associations in an episode are stored together in an auto-associative network that is most likely situated in the hippocampus. Retrieval of inferred associations might therefore occur through hippocampal pattern completion, which is already thought to retrieve encoded associations in episodic memory (Gardner-Medwin, 1976; Marr, 1971; McClelland, 1995; Nakazawa et al., 2002; Wills et al., 2005).
